# Tuberculosis in Infancy and Childhood: Its Pathology, Prevention and Treatment

**Published:** 1909-03

**Authors:** 


**Tuberculosis in Infancy and Childhood : its Pathology, Prevention and Treatment.** By Various Writers. Edited by T. N. Kelynack, M.D. Pp. xiii, 376. London: Baillière, Tindall & Cox. 1908.

The editor believes that the study of tuberculosis in early life, as well as the organisation of ways and means to secure the prevention and arrest of the disease, are but in their beginnings. This handsome volume—the combined work of forty-one contributors—is an expansion of the symposium on the subject which constituted the July number of the *British Journal of Tuberculosis*, 1907. The writers are mostly well-known specialists, who give expression to the best of English and American opinion. Norwegian, Swedish, Swiss and French writers join in the work, so that it may be considered as international, giving everything of the latest as regards the protection of childhood and the solution of the tuberculosis problem. Thirty-nine chapters on as many different topics leave little more to be said as regards our present knowledge. It is not easy to select any one of these for special comment, the work is one for careful study rather than a cursory review, but the differentiation of bovine tuberculosis is, perhaps, one of the most important. On this subject Dr. Nathan Raw sums up his views as follows :—

(1) Tubercle bacilli of **typus humanus** produce pulmonary tuberculosis, tuberculous ulceration of the intestines, tuberculosis of the abdominal glands.

(2) Tubercle bacilli of the **typus bovinus** produce mesenteric tuberculosis, tuberculous peritonitis, tuberculous glands, tuberculous affections of the bones and joints, tuberculous meningitis, acute miliary tuberculosis, and probably lupus.

He further summarises our present position thus:—

(1) Human and bovine tuberculosis are different varieties of a common species.

(2) The human body is susceptible to both forms.

(3) Bovine tuberculosis is frequently conveyed to humans both by means of infected food and by contagion.

(4) These two forms of tubercle are antagonistic to each other.

(5) A mild attack of bovine tuberculosis protects against phthisis pulmonitis.

(6) Tuberculin from human sources has a marked effect on bovine lesions.

(7) Tuberculin from bovine sources has a curative effect on pulmonary tuberculosis.

The Alpine and American stations for tuberculous children are well described, but climates are becoming a secondary consideration.

